# Establishment of Cohesion at the Pericentromere by the Ctf19 Kinetochore Subcomplex and the Replication Fork-Associated Factor, Csm3

**DOI:** 10.1371/journal.pgen.1000629

**Published:** 2009-09-04

**Authors:** Josefin Fernius, Adele L. Marston

**Affiliations:** The Wellcome Trust Centre for Cell Biology, School of Biological Sciences, University of Edinburgh, Edinburgh, United Kingdom; National Cancer Institute, United States of America

## Abstract

The cohesin complex holds sister chromatids together from the time of their duplication in S phase until their separation during mitosis. Although cohesin is found along the length of chromosomes, it is most abundant at the centromere and surrounding region, the pericentromere. We show here that the budding yeast Ctf19 kinetochore subcomplex and the replication fork-associated factor, Csm3, are both important mediators of pericentromeric cohesion, but they act through distinct mechanisms. We show that components of the Ctf19 complex direct the increased association of cohesin with the pericentromere. In contrast, Csm3 is dispensable for cohesin enrichment in the pericentromere but is essential in ensuring its functionality in holding sister centromeres together. Consistently, cells lacking Csm3 show additive cohesion defects in combination with mutants in the Ctf19 complex. Furthermore, delaying DNA replication rescues the cohesion defect observed in cells lacking Ctf19 complex components, but not Csm3. We propose that the Ctf19 complex ensures additional loading of cohesin at centromeres prior to passage of the replication fork, thereby ensuring its incorporation into functional linkages through a process requiring Csm3.

## Author Summary

During cell division, chromosomes must be distributed accurately to daughter cells to protect against aneuploidy, a state in which cells have too few or too many chromosomes, and which is associated with diseases such as cancer and birth defects. This process begins with the generation of an exact copy of each chromosome and the establishment of tight linkages that hold the newly duplicated sister chromosomes together. These linkages, generated by the cohesin complex, are essential to resist the pulling forces of the spindle, which will pull the sister chromosomes apart into the two new daughter cells. Here we examine the establishment of cohesin at the pericentromere, the region surrounding the site of spindle attachment and where its forces are strongest. We find that a dedicated pathway promotes cohesin establishment in this region through a two-step mechanism. In the first step, a group of proteins, known as the Ctf19 complex, promote the association of cohesin with this region. In the second step, the Csm3 protein, which is coupled to the DNA replication machinery, ensures its conversion into functional linkages. We demonstrate the importance of this process for accurate chromosome segregation during cell division.

## Introduction

The accurate transmission of the eukaryotic genome requires that the two copies of each chromosome are held together following their synthesis in S phase until the time of their segregation in mitosis. This chromatid cohesion, which facilitates the biorientation of sister chromatids on the mitotic spindle, is achieved by a multi-subunit complex known as cohesin (reviewed in [1]). Once proper bipolar attachment is achieved, a protease, separase, cleaves the Scc1/Mcd1 subunit of cohesin and destroys the linkages, thereby triggering the movement of sister chromatids to opposite poles [1].

The establishment of cohesion between sister chromatids is coupled to their replication in S phase. In budding yeast, cohesin is loaded onto chromosomes before DNA replication in a manner dependent on, and at the binding sites of, the cohesin-loading complex Scc2/Scc4 [2],[3]. Subsequently, cohesin is thought to translocate from these sites as a result of passage of the transcriptional apparatus [3]. Transformation of this loaded cohesin into functional linkages between sister chromatids requires a second step that takes place during S phase. Scc1 produced after S phase associates with chromosomes but fails to generate cohesion [4]. Several proteins that travel with the replication fork function in this second step. Among the replication fork-associated factors that have been implicated in cohesion function is the Tof1-Csm3 complex which is required for replication fork pausing at replication barriers [5]–[11]. These observations suggest a tight coupling between cohesion establishment and passage of the replication fork.

Analysis of cohesin distribution along both mitotic and meiotic chromosomes of budding yeast has revealed that the highest levels of cohesin are found in a ∼50 kb domain surrounding the ∼120 bp centromere sequence, called the pericentromere [3], [12]–[14]. In fission yeast, pericentromeric heterochromatin is important for cohesin association with the pericentromere during mitosis and meiosis [15]–[18]. Budding yeast lacks pericentromeric heterochromatin but a functional kinetochore is required for pericentromeric cohesin enrichment [13],[19]. The high levels of cohesin in the pericentromere raised a paradox because sister centromeres are known to separate under tension over an approximately 20 kb domain without cohesin cleavage, a phenomenon known as “centromere breathing” [20]–[22]. A possible solution to the paradox was provided by the observation that exertion of tension across sister kinetochores causes a dramatic reduction in the amount of cohesin associated with this 20 kb domain where breathing is observed [19],[23]. However, this region of the pericentromere is thought to form an intramolecular loop, so cohesin may not link sisters in this region [24]. Therefore, although pericentromeric cohesin plays a role in chromosome segregation that cannot be fulfilled by arm cohesins [19], it exhibits unique behavior, the functional relevance of which is unclear.

**Figure 1:**
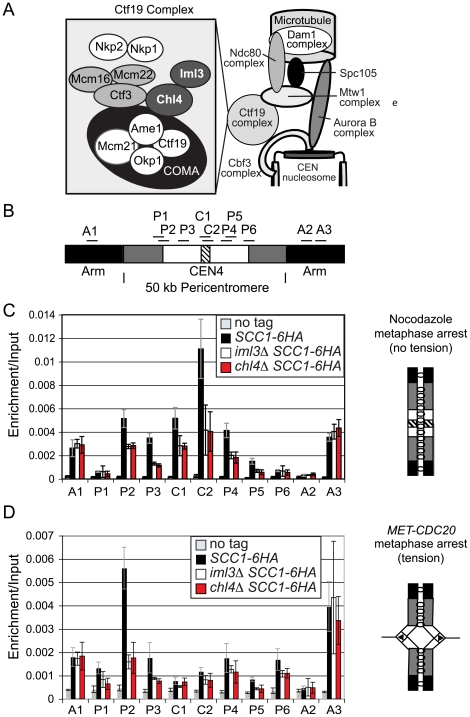
Analysis of Scc1 localization at the pericentromere and the effect of tension in Ctf19 complex mutants. (A) Schematic diagram of the Ctf19 kinetochore subcomplex adapted from [31],[76]. (B) Schematic diagram showing locations of primers used for qPCR analysis of ChIP samples. The hatched area represents the core centromere, and the black, chromosome arms. The ∼50 kb pericentromere is split into white, representing the ∼20 kb region in which cohesin is displaced and sister chromatids separate under tension. The remainder of the pericentromere in which cohesin is retained under tension is shown in grey. (C) Analysis of Scc1-6HA association in cells arrested in metaphase of mitosis in the absence of microtubules. Strains AM1145 (*SCC1-6HA*), AM3441 (*iml3Δ SCC1-6HA*), AM3442 (*chl4Δ SCC1-*6HA), and AM1176 (no tag) were arrested in medium containing nocodazole and benomyl for 3 h to depolymerize microtubules and induce a metaphase arrest. (D) Analysis of Scc1-6HA association in cells arrested in metaphase of mitosis with sister kinetochores under tension. Strains AM1105 (*SCC1-6HA*), AM3948 (*iml3Δ SCC1-6HA*), AM3950 (*chl4Δ SCC1-6HA*), and AM2508 (no tag control) all carrying *MET-CDC20* were arrested in G1 using alpha factor and released into medium containing methionine to deplete *CDC20*. Cells were harvested 2 hours after release from G1 and metaphase arrest confirmed. The mean values from three independent experiments are shown with error bars indicating standard deviation in (C) and (D).

A clear role for pericentromeric cohesin has, however, emerged in meiosis. In contrast to mitosis, where cohesins are removed along the length of chromosomes simultaneously, meiosis requires that loss of arm cohesins and pericentromeric cohesins are temporally separated (reviewed in [25]). The Shugoshin protein (Sgo1) localizes to the ∼50 kb pericentromeric domain of enriched cohesin and protects it from separase-dependent cleavage [14], [26]–[29]. This pericentromeric cohesin is essential for the accurate segregation of sister chromatids during meiosis II. Two kinetochore proteins, Iml3 and Chl4, are important for preventing non-disjunction of sister chromatids during meiosis II [27],[30] and for proper localization of Sgo1, a role shared with cohesin [14]. Iml3 and Chl4 are components of a conserved kinetochore subcomplex, known as the Ctf19 complex (see [31] for review) (Figure 1A). Ctf19 is required for the enhancement of cohesin in the pericentromere [19], but whether this function is shared with other members of the complex was not known. Similarly, Csm3, and its fission yeast counterpart, Swi3, have been implicated in pericentromeric cohesion through screens in both meiosis and mitosis [9]–[11],[32].

Here we further investigate the role of Csm3 and the Ctf19 complex in the establishment of cohesion at the pericentromere. We show that two of the more peripheral Ctf19 complex proteins, Iml3 and Chl4, direct increased cohesin association with the pericentromeric region during both mitosis and meiosis. In the absence of Iml3 and Chl4, cohesin binding at the pericentromere is reduced and this has important implications for sister chromatid cohesion and chromosome segregation. Conversely, we find that the replication-fork associated protein, Csm3, is not required for cohesin association with the pericentromere, but plays a role in cohesion establishment at the pericentromere that is non-overlapping with Iml3 and Chl4. Our results indicate that Iml3 and Chl4 ensure the association of cohesin with the pericentromeric region, to facilitate its subsequent incorporation into functional linkages through a replication-coupled step, which requires Csm3.

## Results

### Iml3 and Chl4 direct increased Scc1 binding at the pericentromere during mitosis

Iml3 and Chl4 are peripheral components of the Ctf19 kinetochore subcomplex [33],[34] (Figure 1A). We asked if Iml3 and Chl4 share the reported role of Ctf19 [19] in promoting the enhancement of cohesin in the pericentromere. We examined the localization of the cohesin subunit, Scc1, at 11 sites on chromosome IV (Figure 1B) by chromatin immunoprecipitation (ChIP) followed by real-time quantitative PCR (qPCR). For the purposes of this paper “centromere” refers to the ∼120 bp region where the kinetochore assembles, “pericentromere” we define as the ∼50 kb enriched cohesin domain and “inner pericentromere” describes the domain in which “breathing” and cohesin removal is observed under tension. We first treated cells with the microtubule-depolymerizing drugs, nocodazole and benomyl, to generate a metaphase arrest where sister kinetochores are not under tension. This has been previously shown to result in high levels of cohesin association with centromeric and pericentromeric regions in wild type cells [19],[23]. In wild type cells (Figure 1C, *SCC1-6HA*, black bars), Scc1 levels were high at cohesin-enriched chromosomal arm sites (A1, A3), but low at the cohesin-poor A2 site. Scc1 association with all pericentromeric sites was increased in wild type cells over the no tag control, although we observed particularly high enrichment at the centromere (C1, C2) and pericentromeric sites P2–4 (Figure 1C). In *iml3Δ* and *chl4Δ* mutants, Scc1 levels at chromosomal arm sites (A1–3) were comparable to that seen in wild type cells (Figure 1C), indicating that Iml3 and Chl4 do not affect cohesin association with chromosome arms. In contrast, Scc1 was reduced at centromeric (C1, C2) and pericentromeric (P2–4) sites and the enrichment over chromosome arms was lost. Similar results were obtained for chromosome V (Figure S1). Therefore, the role of Ctf19 in promoting assembly of a cohesin-rich pericentromeric domain is shared with other components of the complex.

To investigate the effect of tension across sister kinetochores on cohesin association with the pericentromere in cells lacking *IML3* or *CHL4*, we arrested cells by depletion of *CDC20* (under control of the methionine-repressible promoter, *MET-CDC20*; Figure 1D). This generates a metaphase arrest where sister kinetochores attach to opposite poles and are under tension, resulting in a reduction in cohesin levels within the ∼20 kb inner pericentromere [19],[23]. Again Scc1 association on chromosomal arm sites was unchanged in *iml3Δ* or *chl4Δ* mutants (Figure 1D, A1–3). In contrast to the situation in nocodazole (Figure 1C), however, we observed little enrichment of Scc1 at the centromere (C1, C2) or at the majority of pericentromeric sites (P3–6), even in wild type cells (note the scales in Figure 1C and 1D are different). Outside this region, cohesin is retained upon the exertion of tension in wild type cells (site P2). However, we observed a clear reduction in Scc1 association with this site in *iml3Δ* and *chl4Δ* mutants in the presence of tension. This indicates that Iml3 and Chl4 may also restrict the region in the pericentromere where cohesin shows tension-dependent association.

### Defective pericentromeric cohesion in Ctf19 complex mutants

**Figure 2:**
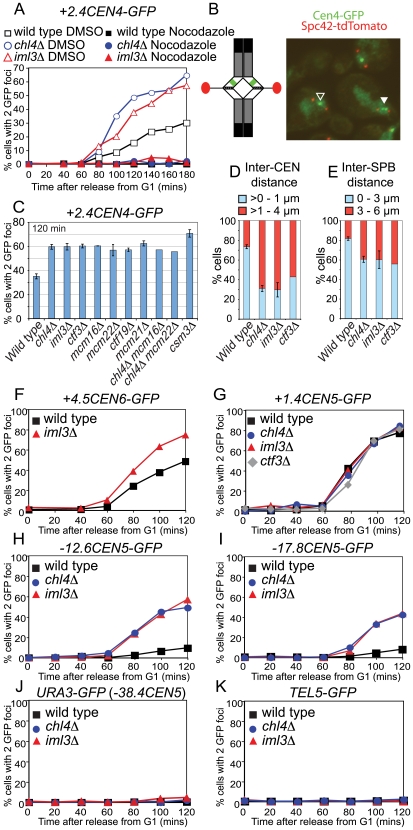
Ctf19 complex mutants exhibit pericentromeric cohesion defects in mitosis. (A) Sister centromeres (*+2.4CEN4-GFP*; 2.4 kb to the right of *CEN4*) are more frequently separated in a metaphase arrest in *iml3Δ*and *chl4Δ* mutants and this depends on microtubules. Strains of wild type (AM914), *iml3Δ* (AM3522), and *chl4Δ* (AM3501), all carrying *+2.4CEN4-GFP* (2.4 kb to the right of *CEN4*) and *MET-CDC20* were arrested in alpha factor and then released into medium containing methionine to deplete *CDC20* and either DMSO (control) or nocodazole (to depolymerize microtubules). Samples were taken at the indicated times after release from G1 for anti-tubulin immunofluorescence (Figure S2) and to determine the number of GFP foci per nucleus. Percentages of cells with two GFP dots are shown for wild type (black squares), *chl4Δ* (blue circles), and *iml3Δ* (red triangles) treated with either DMSO (open symbols) or nocodazole (closed symbols). 200 cells were scored at each time point. (B) Cohesion defects were examined after releasing cells carrying *+2.4CEN4-GFP*, *SPC42-tdTomato* (to label spindle pole bodies) and *MET-CDC20* from a G1 block, as a result of alpha factor treatment, into medium containing methionine to deplete *CDC20*. A snapshot of cells at the 120 min time point (metaphase arrest) is shown as an example together with a schematic diagram of chromosome IV with sister centromeres under tension (left). SPBs are shown in red and *+2.4CEN4-GFP* is shown in green. The black and white arrowheads indicate cohesed and separated *+2.4CEN4-GFP* foci, respectively. (C) Summary of frequency of *+2.4CEN4-GFP* separation at metaphase in Ctf19 complex mutants. Values are the mean of all experiments where percentages of separated sister centromeres were determined after scoring 200 cells, 120 min after release from G1, in a metaphase arrest. Error bars indicate standard error. Strains, and number of independent repeats (given in italics), were: wild type (AM4643; *14*), *chl4Δ* (AM4644; *10*), *iml3Δ* (AM4647; *7*), *ctf3Δ* (AM4683; *3*), *mcm16Δ* (AM6158; *2*), *mcm22Δ* (AM6160; *2*), *ctf19Δ* (AM5786; *3*), *mcm21Δ* (AM5788; *3*), *chl4Δ mcm16Δ* (AM6195; *1*), *chl4Δ mcm22Δ* (AM6192, *1*), and *csm3Δ* (AM4717; *6*). (D,E) Sister centromeres and SPBs are further apart at metaphase in *iml3Δ*, *chl4Δ*, and *ctf3Δ* mutants. The distance between *+2.4CEN4-GFP* (D) and *SPC42-tdTomato* (E) foci was determined for all separated foci from the 60–120 minute time points. For wild type (AM4643), *chl4Δ* (AM4644), and *iml3Δ* (AM4647), the average of two independent experiments with error bars representing standard deviation is shown. For *ctf3Δ* (AM4683), measurements are from a single experiment. (D) The percentage of *+2.4CEN4-GFP* foci that were >0–1 µm (blue) or >1–4 µm (red) apart. *n = *115/82 (wild type); 202/139 (*chl4Δ*), 184/167 (*iml3Δ*); 199 (*ctf3Δ*). (E) The percentage of *SPC42-tdTomato* foci that were >0–3 µm (blue) or >3–6 µm (red) apart is shown. *n = *338/305 (wild type); 548/244 (*chl4Δ*), 331/272 (*iml3Δ*); 313 (*ctf3Δ*). The percentages of separated spindle pole bodies or *+2.4CEN4-GFP* foci are shown in Figure S2 for this experiment. (F–K) The cohesion defect in *iml3Δ* and *chl4Δ* mutants is not chromosome-specific but is restricted to centromere-proximal regions. Cells of the indicated genotypes carrying *MET-CDC20*, *SPC42-tdTomato* and with GFP labels at various loci were arrested in G1 with alpha factor and released into medium containing methionine and the percentages of separated GFP foci were scored at the indicated timepoints. *tet* operators are integrated at: (F) *+4.5CEN6-GFP* (4.5 kb to right of *CEN6*) in wild type (AM5329) and *iml3Δ* (AM5330); (G) *+1.4CEN5-GFP* (1.4 kb to right of *CEN5*) in wild type (AM5189), *iml3Δ* (AM5249), *chl4Δ* (AM5251), and *ctf3Δ* (AM5188); (H) *-12.6CEN5-GFP* (12.6 kb to left of *CEN5*) in wild type (AM5545), *iml3Δ* (AM5542), and *chl4Δ* (AM5560); (I) *-17.8CEN5-GFP* (17.8 kb to left of *CEN5*) in wild type (AM5533), *iml3Δ* (AM5537) and *chl4Δ* (AM5551); (J) *URA3-GFP* (38.4 kb to left of *CEN5*) in wild type (AM1081) and *iml3Δ* (AM3541) and *chl4Δ* (AM3519). (K) *-30RTEL5* (∼30 kb from telomere on right arm of chromosome V) in wild type (AM2511) and *iml3Δ* (AM3887) and *chl4Δ* (AM3942). Note that the *URA3-GFP* and *TEL5-GFP* strains did not carry *SPC42-tdTomato*. Distances indicate the start of the *tetO* array from the centromere.

We investigated the importance of pericentromeric Scc1 for sister chromatid cohesion during mitosis by inducing a metaphase arrest in strains with *tetO* arrays integrated 2.4 kb from the centromere of chromosome IV and which express TetR-GFP (*+2.4CEN4-GFP*; [21]). Previous reports have indicated that microtubule forces cause sister centromeres to separate specifically at inner pericentromeric regions during metaphase, so that 2 GFP dots are observed in a fraction of wild type cells [20]–[22]. Wild type, *iml3Δ* and *chl4Δ* cells were released from a G1 block into a metaphase arrest by depletion of *CDC20*, either in the presence of nocodazole (in DMSO) to depolymerize microtubules or DMSO alone. Depletion of *CDC20* caused a metaphase arrest in all three strains, as judged by spindle morphology in the strains treated with DMSO (Figure S2A) or nuclear morphology in the nocodazole-treated strains (not shown). We scored separation of sister chromatids at *CEN4* by monitoring the numbers of cells in which 2 GFP dots were visible (Figure 2A). Consistent with previous reports [20]–[22], we observed separation of sister *+2.4CEN4-GFP* in ∼30% of wild type cells in a manner dependent on microtubules (Figure 2A). This fraction was increased to ∼60% in *iml3Δ* and *chl4Δ* mutants and was also microtubule-dependent (Figure 2A). This indicates that Iml3 and Chl4 are required to generate cohesion at the pericentromere that is effective in resisting microtubule pulling forces.

### Ctf19 complex mutants exhibit non-equivalent chromosome loss rates but similar cohesion defects

Ctf19 complex components are required for accurate chromosome transmission during mitosis [33], [35]–[40]. We used a colony-sectoring assay [41] to measure chromosome loss in mutants lacking Ctf19 complex components or Csm3. All mutants tested, with the exception of *nkp1Δ* and *nkp2Δ*, exhibited chromosome loss rates that were elevated compared to the wild type, however the frequencies were not equivalent (Table S1). The highest chromosome loss rates were observed in *mcm21Δ* and *ctf19Δ* mutants, with other mutants exhibiting lower, but non-equivalent rates of loss.

We compared cohesion defects in Ctf19 complex mutants by examining the separation of *+2.4CEN4-GFP* foci in cells arrested in metaphase by *CDC20* depletion. Figure 2C shows that 120 mins after release from G1, GFP foci separation was similarly increased over wild type in all single and double mutant combinations tested (see also representative time courses Figure S3), despite their different chromosome loss rates. Previous analyses have revealed that the association of Iml3, Chl4, Ctf3, Mcm16 and Mcm22 with kinetochores depends on Ctf19, but not *vice-versa* [33],[39]. We found that Chl4 also fails to associate with *CEN4* in a *mcm21Δ* mutant and its levels are reduced in both *mcm16Δ* and *iml3Δ* mutants (Figure S4). These findings suggest that Ctf19 and Mcm21 could mediate cohesion establishment through recruitment of the more peripheral components of the sub-complex.

### Increased distance between sister centromeres in Ctf19 complex mutants

We next asked if the distance between the separated centromeres is increased in *iml3Δ, chl4Δ* and *ctf3Δ* mutants arrested in metaphase by depletion of *CDC20*. An *SPC42-dtTomato* construct was used to visualize spindle pole bodies (SPBs) together with *+2.4CEN4-GFP* (Figure 2B) and directly monitor the accumulation of cells in metaphase (Figure S2B) alongside sister centromere separation (Figure S2C). Measurement of distances between separated *+2.4CEN4-GFP* foci from the 60–120 min time points revealed that the majority of separated sister centromeres were greater than 0 but less than 1 µm apart in wild type cells, consistent with previously published observations (Figure 2D) [21]. However, in *iml3Δ*, *chl4Δ* and *ctf3Δ* mutants, most of the separated sister centromeres were greater than 1 but less than 4 µm apart. Perhaps as a consequence of this increased centromere stretching, we found that the distance between SPBs was also increased in *iml3Δ, chl4Δ* and *ctf3Δ* mutants compared to wild type cells (Figure 2E). We conclude that both the frequency and distance of sister centromere separation is increased at metaphase in the absence of Ctf19 complex components.

### The Ctf19 complex restricts the domain around the centromere at which sister chromatids separate at metaphase

To ask if the cohesion defects observed in Ctf19 complex mutants at *CEN4* also apply to other centromeres we used GFP labels in which *tetO* arrays are integrated 4.5 kb from *CEN6* (Figure 2F) or 1.4 kb from *CEN5* (Figure 2G; [22]). Figure 2F shows that *CEN6-GFP* behaves in a manner reminiscent of *+2.4CEN4-GFP* in both wild type and *iml3Δ* cells. That is, sister centromeres separated at an appreciable frequency in wild type cells (∼50%), but with a greater occurrence (∼75%) in the *iml3Δ* mutant cells. *CEN5-GFP*, however, exhibited a different behavior, because sister centromere separation was nearly complete (∼80%) in wild type metaphase-arrested cells, perhaps as a result of the close proximity of this label to the centromere [2] and no increase in *CEN5-GFP* separation was observed in *iml3Δ*, *chl4Δ* or *ctf3Δ* mutant cells (Figure 2G). Because the Ctf19 complex is required for normal levels of the cohesin Scc1 at the pericentromere of chromosome V during mitosis (Figure S1) we reasoned that cohesion defects might be apparent at loci more distant from *CEN5*. Indeed, two intermediate sites in the pericentromere, at ∼12.6 kb and ∼17.8 kb to the left of *CEN5* (*-12.6CEN5-GFP* and *-17.8CEN5-GFP*, respectively), which split rarely in wild type cells [21], showed an increased separation in the absence of *IML3* or *CHL4* (Figure 2H and 2I). However, cohesion at a site just outside the pericentromere (*URA3-GFP*; 38.4 kb from *CEN5* [42]) was virtually unaffected in *iml3Δ* and *chl4Δ* mutants (Figure 2J) and splitting of GFP signals at a telomeric locus on chromosome V (*TEL5-GFP*) was not observed in any of the strains (Figure 2K). This is consistent with our ChIP results and indicates that arm cohesion is intact in *iml3Δ* and *chl4Δ* mutants. Our results suggest that the Ctf19 complex directs cohesion establishment at the pericentromere of budding yeast chromosomes and that this is essential to restrict the region where sister chromatids separate when their kinetochores are under tension.

### The Ctf19 complex also directs cohesion establishment during meiosis

**Figure 3:**
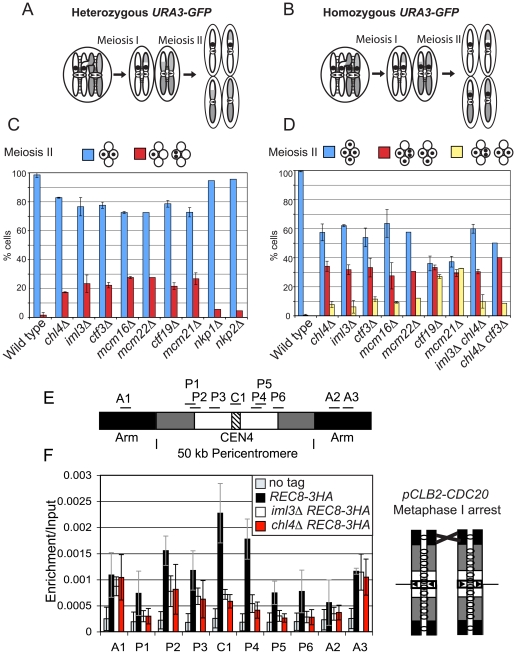
The Ctf19 complex promotes cohesion establishment in the pericentromere during meiosis. Schematic diagram showing the segregation of chromosome V with either heterozygous (A) or homozygous (B) GFP labels in wild type cells. Homologs are shown in white and grey and the GFP dot is shown as a black circle. Sister chromatid cohesion is represented by black lines between sister chromatids. Arrows on kinetochores indicate the direction of attachment to the spindle. (C,D) Ctf19 complex mutations lead to errors in chromosome segregation during meiosis. Cells of the indicated genotypes in which either one copy [(C); heterozygous) or both copies (D); homozygous] of chromosome V were marked with GFP (*tetR-GFP*::*LEU2 URA3::tetOx224*) at *URA3* (38.4 kb from the centromere) were induced to sporulate at 30°C. The percentage of tetranucleate cells with the patterns of GFP dot segregation shown was determined after examining 200 cells, 8 hours after transfer into sporulation medium. Strains used were AM107 (wild type), AM1904 (*iml3Δ*), AM1902 (*chl4Δ*), AM5104 (*ctf3Δ*), AM5105 (*mcm16Δ*), AM3684 (*mcm22Δ*), AM5107 (*ctf19Δ*), AM436 (*mcm21Δ*), AM4781 (*nkp1Δ*), and AM4988 (*nkp2Δ*) for (C) and AM1603 (wild type), AM1903 (*iml3Δ*), AM1905 (*chl4Δ*), AM3811 (*iml3Δ chl4Δ*), AM4059 (*ctf3Δ*), AM3799 (*mcm16Δ*), AM3661 (*mcm22Δ*), AM3798 (*ctf19Δ*), AM437 (*mcm21Δ*), and AM5809 (*chl4Δ ctf3Δ*) for (D). Values shown are the mean from two independent experiments, with error bars representing standard deviation, with the exception of *mcm22Δ*, *nkp1Δ*, *nkp2Δ* (C) and *mcm22Δ* and *chl4Δ ctf3Δ* (D), where results are shown from a single experiment. (E,F) Reduced levels of the meiotic cohesin, Rec8, at the pericentromere in Ctf19 complex mutants. (E) Schematic diagram of the primer sets used for qPCR on chromosome IV. (F) Analysis of Rec8-3HA association in cells arrested in metaphase I in meiosis. Strains AM3560 (no tag control), AM3375 (*REC8-3HA*), AM3422 (*chl4Δ REC8-3HA*) and AM3377 (*iml3Δ REC8-3HA*) carrying the *pCLB2-3HA-CDC20* allele were harvested for ChIP 8 h after transfer into sporulation medium. The schematic diagram shows the configuration of the chromosomes. qPCR analysis of anti-HA ChIP samples was performed using primers shown in (E).

Pericentromeric cohesion is particularly important during meiosis. Following the segregation of homologous chromosomes during meiosis I, sister chromatids lack all arm cohesion and are solely reliant on pericentromeric cohesion. This property of meiosis exposes weak general cohesion defects [43] and also led us to identify *IML3* and *CHL4* as important mediators of pericentromeric cohesion [27]. To assess the importance of other Ctf19 complex components for meiosis II, we examined the fate of a GFP label at *URA3* (38.4 kb from *CEN5*) on either one or both copies of chromosome V (heterozygous or homozygous GFP dots, Figure 3A and 3B, respectively) after meiosis. Examination of the fate of heterozygous dots after meiosis II revealed that sister GFP labels failed to segregate into different nuclei in approximately 20% of tetranucleate cells of all Ctf19 complex mutants tested, with the exception of *nkp1Δ* and *nkp2Δ* (Figure 3C). Meiosis II non-disjunction in Ctf19 complex mutants with homozygous GFP dots was also evident from the high frequency of tetranucleate cells with a GFP dot in just 3 of the 4 spores, which was comparable also in *iml3Δ chl4Δ* or *chl4Δ ctf3Δ* double mutant combinations (Figure 3D). Although most of the mutants with homozygous GFP dots showed only the expected modest number of tetranucleate cells with GFP label in just two spores, *ctf19Δ* and *mcm21Δ* mutants were a notable exception (Figure 3D). Examination of GFP dot segregation in binucleate cells (Figure S5) revealed that Ctf19 and Mcm21, but not other Ctf19 complex components, are also required for accurate segregation during meiosis I. These findings are consistent with our conclusion from the mitotic chromosome loss data (Table S1) that Ctf19 and Mcm21 promote accurate chromosome segregation also in ways other than cohesion establishment.

To address whether meiosis II non-disjunction in Ctf19 complex mutants could be due to a failure to enrich cohesin in the pericentromere, as in mitosis, we examined localization of the meiosis-specific counterpart of Scc1, Rec8, during meiosis. Wild type, *iml3Δ* and *chl4Δ* cells were arrested in metaphase I (by depletion of *CDC20* [44]), and the localization of the meiotic cohesin, Rec8, was examined at 10 sites on chromosome IV (Figure 3E) by ChIP followed by qPCR. Because sister kinetochores are uniquely mono-oriented during meiosis I (Figure 3F), this leads to a situation where they are not under tension, enabling the retention of high levels of cohesin in the centromere and pericentromere. Consistent with previous reports, Rec8 localization closely resembled that of the mitotic cohesin, Scc1, in wild type cells [12],[14]. As in mitosis, deletion of *IML3* or *CHL4* caused a reduction in Rec8 levels at the centromere (C1) and pericentromeric sites (P1–6), but not at chromosomal arm sites (A1–3). We also observed a similar requirement for Ctf19 in localizing Iml3 and Chl4 during meiosis, and a partial dependence on Mcm16 and Mcm22 (Figure S6). Furthermore, we found that Iml3 and Chl4 are localized specifically at the core centromere, despite their ability to influence cohesin in the surrounding pericentromere (Figure S6). We conclude that the Ctf19 complex plays similar roles in pericentromeric cohesion establishment during mitosis and meiosis.

### Non-overlapping roles of Csm3 and the Ctf19 complex in cohesion

**Figure 4:**
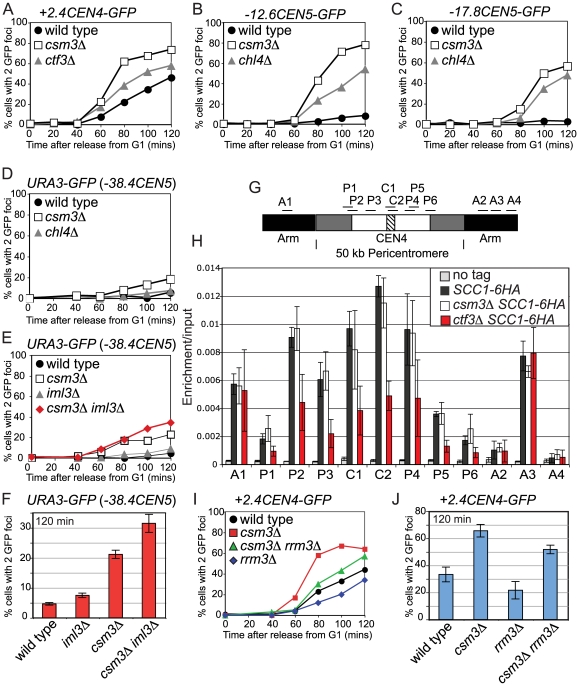
Csm3 promotes cohesion establishment at the pericentromere but is not required for cohesin enrichment in this region. (A–E) *csm3Δ* mutants show cohesion defects in mitosis that are additive with *iml3Δ*. Strains carrying the indicated GFP labels and *MET-CDC20* were released from G1 into a metaphase arrest by *CDC20* depletion, and the number of GFP foci per nucleus was determined for 200 cells. Strains with *+2.4CEN4-GFP*, *-12.6CEN5-GFP*, and *-17.8CEN5-GFP* also carried *SPC42-tdTomato.* (A) Separation of *+2.4CEN4-GFP* foci in wild type (AM4643), *csm3Δ* (AM4717), and *ctf3Δ* (AM4683). (B) Separation of *-12.6CEN5-GFP* foci in wild type (AM5545), *csm3Δ* (AM5569), and *chl4Δ* (AM5560). (C) Separation of *-17.8CEN5-GFP* foci in wild type (AM5533), *csm3Δ* (AM5564), and *chl4Δ* (AM5551). (D) Separation of *URA3-GFP* foci in wild type (AM1081), *chl4Δ* (AM3519), and *csm3Δ* (AM5312). (E) Separation of *URA3-GFP* foci in wild type (AM1081), *iml3Δ* (AM3541), *csm3Δ* (AM5312), and *csm3Δ iml3Δ* (AM5796). (F) Summary of frequency of *URA3-GFP* foci separation at the 120 min timepoint. Values are the mean from 3 (wild type, *iml3Δ*, *csm3Δ*) or 2 (*iml3Δ csm3Δ*) experiments with error bars indicating standard error. For each experiment 200 cells were scored. (G,H) Csm3 is not required for cohesin enrichment in the pericentromere. Strains AM1145 (*SCC1-6HA*), AM4927 (*ctf3Δ SCC1-6HA*), AM3757 (*csm3Δ SCC1-*6HA), and AM1176 (no tag) were arrested in medium containing nocodazole and benomyl for 3 h to depolymerize microtubules and induce a metaphase arrest. Primers at locations depicted in (G) were used for analysis of Scc1-6HA association by qPCR after ChIP (H). (I,J) Deletion of *RRM3* can partially rescue the cohesion defect of *csm3Δ* mutants. Separation of *+2.4CEN4-GFP* foci in wild type (AM4643), *csm3Δ* (AM4717), *rrm3Δ* (AM6068), and *csm3Δ rrm3Δ* (AM6066) in a representative experiment (I) and mean values at 120 mins from 3 independent experiments with error bars indicating standard error (J).

To understand more about cohesion establishment in the pericentromere, we extended our analysis to Csm3, which is required for proper chromosome segregation in meiosis [10] and has been implicated in cohesion establishment during mitosis [9],[11]. Furthermore, Swi3, the fission yeast homolog of Csm3, was shown to have a role in cohesion establishment [32]. Examination of *+2.4CEN4-GFP* separation in *csm3Δ* cells arrested in metaphase by *CDC20* depletion confirmed that *csm3Δ* mutants exhibit a cohesion defect that is more severe than that of Ctf19 complex mutants (Figures 2C and 4A). Consistently, *csm3Δ* cells showed a more pronounced cohesion defect than *chl4Δ* cells at loci 12.6 and 17.8 kb away from *CEN5* (*-12.6CEN5-GFP* and *-17.8CEN5-GFP*; Figure 4B and 4C, respectively). Furthermore, in contrast to *chl4Δ* mutants and in agreement with previous reports [9],[11], cells lacking *CSM3* exhibited a small defect in cohesion at the *URA3-GFP* locus (Figure 4E). Examination of *URA3-GFP* separation in *csm3Δ iml3Δ* double mutants, revealed an additive effect (Figure 4E and 4F). Similarly, we observed additive effects on meiotic chromosome segregation in *csm3Δ iml3Δ* mutants (Figure S7). These results indicate that Csm3 plays a role in cohesion establishment that is non-overlapping with the Ctf19 complex and which may not be restricted to the pericentromere.

### Csm3 acts after cohesin loading and may couple cohesion establishment to replication fork progression

To ask if the cohesion defects of *csm3Δ* mutants could, like that of Ctf19 complex mutants, be due to a failure to recruit proper levels of cohesin to chromosomes, we examined the localization of Scc1 in nocodazole-arrested *csm3Δ* mutants (Figure 4G and 4H). However, we found that Scc1 levels in the *csm3Δ* mutant were comparable to wild type at all sites tested, unlike a Ctf19 complex mutant control, *ctf3Δ* (Figure 4H). This indicates that Csm3 does not promote cohesion establishment by directing cohesin association with chromosomes.

Csm3 is part of a complex with Tof1 that travels with the replication fork and is required for fork stalling at DNA-protein barriers, including centromeres [5]–[8]. Csm3 is thought to achieve fork stalling by counteracting the Rrm3 helicase and deletion of *RRM3* restores fork stalling in *csm3Δ* mutants at replication termination sites [45]. If Csm3 facilitates cohesion establishment by promoting fork stalling, we reasoned that the cohesion defect might also be rescued in *csm3Δ rrm3Δ* mutants. Indeed, deletion of *RRM3* in *csm3Δ* mutants reduced the frequency of *+2.4CEN4-GFP* separation at metaphase (Figure 4I and 4J), although not to wild type levels. Interestingly, the *rrm3Δ* single mutant exhibited a lower frequency of GFP dot separation that wild type (Figure 4I and 4J). Since *rrm3Δ* mutants exhibit increased fork stalling, particularly at hard to replicate sites such as centromeres [46], these results are consistent with the idea that fork stalling facilitates cohesion establishment.

### Delayed and inefficient loading of cohesin at centromeres in *chl4Δ* mutants

**Figure 5:**
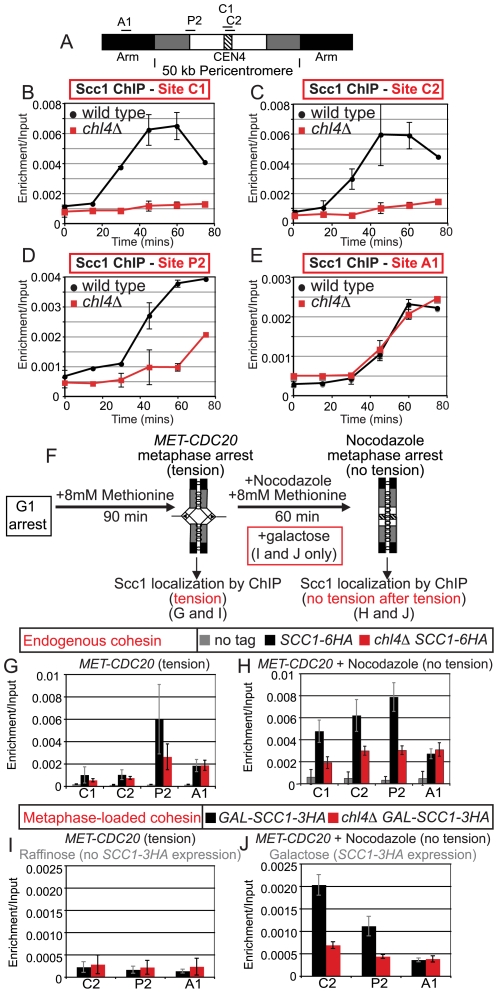
Chl4 directs cohesin association with the pericentromere throughout the cell cycle. (A–E) Time course of cohesin loading in the cell cycle. Wild type (AM4226) and *chl4Δ* (AM4291) strains carrying *MET-CDC20* and *SCC1-6HA* were arrested in G1 with alpha factor then released into medium containing methionine and nocodazole at 18°C. Samples were taken at the indicated timepoints and Scc1-6HA association was analyzed by ChIP and qPCR. (A) Schematic diagram of sites used for qPCR. Results for centromeric sites C1 (B), C2 (C), pericentromeric site P2 (D), and arm site A1 (E) are shown. Where error bars are present, the mean of 3 (C,E) or 2 (B,D) independent experiments are shown with error bars representing standard deviation. Time points without error bars where taken in only one of the experiments. (F–J) Chl4 is required for Scc1 to associate efficiently with the pericentromere during metaphase after the eradication of tension. (F) Schematic diagram showing the experimental set-up. (G,H) Strains AM1105 (*SCC1-6HA*), AM3950 (*chl4Δ SCC1-6HA*), and AM2508 (no tag control), carrying *MET-CDC20* were arrested in G1 and then released into medium containing methionine for 90 min to induce a metaphase arrest in the presence of microtubules. Half the culture was harvested for Scc1-6HA ChIP analysis and nocodazole was added to the remainder to depolymerize microtubules. After 60 min the nocadazole-treated sample was harvested for ChIP analysis. (G). qPCR analysis after ChIP prior to nocodazole addition. (H) qPCR analysis after ChIP following microtubule depolymerization. (I,J) Chl4 contributes to the *de novo* loading of cohesin at the centromere during a metaphase arrest. Strains AM4084 (*pGAL-SCC1-3HA*) and AM5974 (*chl4Δ pGAL-SCC1-3HA*) were treated as described in (G,H) except that cells were arrested in metaphase by *CDC20* depletion in raffinose, rather than glucose medium, and galactose was added to half of the culture, together with nocodazole, to induce *SCC1-3HA* expression. (I) qPCR analysis after ChIP prior to nocodazole and galactose addition. (J) qPCR analysis after ChIP following microtubule depolymerization and *SCC1-3HA* induction.

Next we investigated the role of the Ctf19 complex in pericentromeric cohesin recruitment in more detail. Our results show that while cohesin is reduced at the pericentromere of *iml3* ***Δ*** and *chl4* ***Δ*** cells, a low level remains during a metaphase arrest in the absence of tension (Figure 1C). Because cohesin can associate with chromosomes after S phase, but is not normally cohesive [4], we analyzed the kinetics of cohesin loading in *chl4* ***Δ*** cells in a synchronized mitotic cell cycle. Wild type and *chl4* ***Δ*** cells carrying *MET-CDC20* and *SCC1-6HA* were released from a G1 arrest into methionine and nocodazole at 18°C and samples were taken at 15 min intervals for analysis of Scc1 localization at 4 sites on chromosome IV (Figure 5A). FACS analysis indicated that both strains entered into S phase after 60 min under these conditions (Figure S8). In wild type cells, cohesin associated with the two centromeric (C1 and C2) sites after only 30 min (Figure 5B and 5C). Cohesin began to be recruited to the chromosome arm (A1) and pericentromeric (P2) sites a little later, at 45 min, in wild type cells (Figure 5D and 5E). The early loading of cohesin at centromeres is consistent with a specialized mechanism for cohesin recruitment operating in this region. In *chl4* ***Δ*** cells, cohesin loaded onto the chromosome arm (A1) site with the same kinetics as in wild type cells (Figure 5E), but was barely detectable at the two centromeric (C1 and C2) sites, even after 75 min (Figure 5B and 5C). Cohesin recruitment was also both delayed and inefficient at the pericentromeric (P2) site in *chl4* ***Δ*** mutants (Figure 5D). These findings suggest that Chl4 is required for the loading of cohesin at the centromere. Furthermore, the almost complete absence of cohesin at centromeres as *chl4Δ* cells enter S phase explains why cohesion establishment fails, given that centromeres replicate early in S phase [47]. It follows that the low, but appreciable, levels of pericentromeric cohesin observed in nocodazole-arrested *chl4* ***Δ*** mutants (Figure 1C) must arrive after passage of the replication fork and therefore may not be functional in holding sister chromatids together.

### Chl4 directs cohesin enrichment at the pericentromere during metaphase

Previous observations have indicated that a pathway that promotes cohesin loading at the centromere might be particularly active in mitotic cells, although the significance of this is unknown [19],[23]. Furthermore, the removal of microtubule forces in metaphase-arrested cells allows cohesin to re-associate with the pericentromere apparently independently of the Scc2/4 complex that is normally responsible for the loading of cohesin at sites throughout the genome [23]. To ask if the Ctf19 complex directs the accumulation of cohesin at the pericentromere during metaphase, we arrested wild type and *chl4* ***Δ*** cells in metaphase with sister kinetochores under tension by depleting *CDC20* and examined the association of Scc1 with sites on chromosome IV (Figure 5F). As before, the presence of tension caused cohesin levels to be very low at the centromere in both strains, similarly high at a chromosomal arm site (A1) and enriched only in the presence of Chl4 at a pericentromeric site (P2) just outside the tension-sensitive region (Figure 5G). We then treated these cells with nocodazole to depolymerize microtubules, thereby eliminating tension, and re-examined cohesin association with these sites (Figure 5H). As expected, cohesin levels did not increase greatly over background upon the eradication of tension at the chromosomal arm site (A1) or the pericentromeric site (P2) in either wild type or *chl4* ***Δ*** mutant cells (Figure 5H). However, cohesin levels increased substantially at both centromeric sites (C1 and C2) in wild type cells after microtubule depolymerization (Figure 5H). In contrast, cohesin accumulation at the centromere during metaphase was more modest in *chl4* ***Δ*** mutants and did not show enrichment over the chromosomal arm site (Figure 5H).

To distinguish between *de novo* loading of cohesin at the pericentromere and redistribution of cohesin from other chromosomal sites, we restricted our analysis to cohesin that was produced only in metaphase. We used *MET-CDC20*-containing strains carrying *SCC1-3HA* under control of the galactose promoter (*pGAL-SCC1-3HA*) but with untagged endogenous cohesin. Cells were released from G1 in the presence of raffinose (to prevent *pGAL* induction) and methionine (to deplete *CDC20*) to allow cells to accumulate in metaphase with endogenous (untagged) cohesin and sister kinetochores under tension. After 90 min, anti-HA ChIP was performed to determine the background signal in the absence of *SCC1-3HA* expression (Figure 5I). Subsequently, nocodazole and galactose were added to depolymerize microtubules and induce expression of *SCC1-3HA*. After a further 60 min, association of Scc1-3HA at 3 chromosomal sites was analyzed. Figure 5J shows that, in wild type cells, only a low level of metaphase-produced cohesin associated with a chromosomal arm site, whereas levels at pericentromeric and centromeric sites were elevated well above background. This is consistent with a previous report [23] showing that *de novo* produced cohesin preferentially associates with the pericentromere during metaphase. In the *chl4* ***Δ*** mutant, however, metaphase-produced cohesin at the C2 and P2 sites was hardly elevated above its levels at the A1 arm site (Figure 5J). These results indicate that Chl4 contributes to the mechanism that drives cohesin loading at the pericentromere during metaphase, although the purpose and functionality of this cohesin remains unknown.

### An S phase delay restores cohesion in *iml3Δ* and *chl4Δ* mutants, but not *csm3Δ* mutants

**Figure 6:**
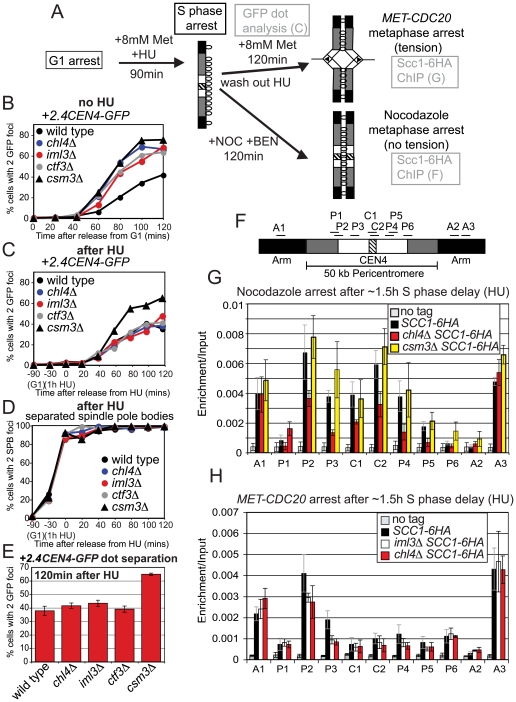
Delaying DNA replication by hydroxyurea treatment rescues the cohesion defect of *iml3Δ* and *chl4Δ*, but not *csm3Δ*, mutants. The experimental setup is shown in (A). Cells were released from G1 into medium containing 8 mM methionine in the presence of HU. After 1.5 h, the HU was washed out and a metaphase arrest was induced either by depletion of *CDC20* or by nocodazole treatment. (B–E) Analysis of *+2.4CEN4-GFP* foci separation after the HU-induced replication delay. Wild type (AM4643), *chl4Δ* (AM4644), *iml3Δ* (AM4647), *ctf3Δ* (AM4683), and *csm3Δ* (AM4717) strains were arrested in G1 with alpha factor, the cultures were split and released into 8 mM methionine either in the absence (B) or presence (C,D) of HU. Samples were taken at the indicated timepoints after release from G1 for scoring of *+2.4CEN4-GFP* (B,C) or *SPC42-tdTomato* (D) separation. FACS analysis was used to confirm a delay in bulk DNA replication in the HU-treated strains (Figure S9). (E) Mean GFP dot separation of 3 experiments at the 120 min time point after washing out HU. Error bars represent standard error. (F–H) Analysis of Scc1-6HA association with chromosome IV during metaphase with sister kinetochores not under tension, after an S phase delay. (F) Location of primer sets used for qPCR analysis. (G) Localization of Scc1 in the absence of microtubules. Strains used were AM1145 (*SCC1-6HA*), AM 3442 (*chl4Δ SCC1-6HA*), AM3757 (*csm3Δ SCC1-6HA*), and AM1176 (no tag). (H) Localization of Scc1 when sister kinetochores are under tension. qPCR analysis of Scc1-6HA ChIP from cells harvested 2 h after HU wash-out. Strains used all carried *MET-CDC20* and were AM1105 (*SCC1-6HA*), AM3948 (*iml3Δ SCC1-6HA*), AM3950 (*chl4Δ SCC1-6HA*), and AM2508 (no tag control).

Next we investigated the relationship between Ctf19 complex-dependent recruitment of cohesin to the pericentromere and DNA replication. Our findings indicate that cohesin is barely above background at the centromere prior to the initiation of DNA replication in *chl4Δ* mutants (Figure 5B and 5C), however, during a metaphase arrest without microtubules a low, but appreciable, level of cohesin accumulates in this region (Figure 1C). If cohesion establishment at the pericentromere occurs through the normal replication-coupled mechanism in S phase, then the late arrival of cohesin to this region in *chl4Δ* cells would exclude its conversion into functional inter-sister linkages. We reasoned that delaying replication fork passage in Ctf19 mutants might allow this late-arriving cohesin to be converted into functional cohesion. The experimental set-up is shown in Figure 6A. We released wild type, *chl4Δ*, *iml3Δ*, *ctf3Δ* and *csm3Δ* cells carrying *MET-CDC20* from a G1 arrest into medium containing hydroxyurea (HU) to inhibit DNA replication and containing 8 mM methionine to deplete *CDC20*. After 90 min in the presence of HU, the drug was washed out and cells were allowed to accumulate in metaphase (as a result of *CDC20* depletion). FACS analysis confirmed an approximately 90 min delay in bulk DNA replication in all strains (Figure S9). As expected, strains activated the DNA damage checkpoint kinase, Rad53 in HU [48], as evidenced by its hyperphosphorylation (Figure S10A), but the slowest migrating forms disappeared rapidly upon release in all strains, although in the case of the *csm3Δ* there was a slight delay (Figure S10A). Nevertheless, none of the mutants showed sensitivity to HU (Figure S10B), indicating that they are able to respond to and recover from the damage caused. In control cells that were not treated with HU, we observed a cohesion defect at *CEN4*, in *chl4Δ*, *iml3Δ*, *ctf3Δ* and *csm3Δ* mutants, as before (Figure 6B). However, remarkably, delaying cells in S phase by HU treatment reduced the separation of sister *+2.4CEN4-GFP* foci to near wild type levels in *iml3Δ*, *chl4Δ* and *ctf3Δ* mutants, but not *csm3Δ* mutants (Figure 6C). In the HU-treated cells, all mutants separated SPBs with approximately the same kinetics (note that SPB duplication is not prevented by HU treatment) (Figure 6D). Therefore, a HU-induced S phase delay rescues the pericentromeric cohesion defect of *iml3Δ* and *chl4Δ* and *ctf3Δ* mutants, but not *csm3Δ* mutants.

We examined the association of Scc1 with chromosomes in wild type, *chl4Δ* and *csm3Δ* cells arrested in metaphase in the absence of microtubules, following a HU-induced S phase delay. Consistent with our finding that Csm3 does not influence cohesin levels in the pericentromere at metaphase in the absence of an S phase delay (Figure 4H), we observed similar levels of cohesin in *csm3Δ* and wild type cells (Figure 6G) after a HU delay. In the absence of *CHL4*, after the HU delay, cohesin levels were, however, reduced in the centromere and pericentromere to a similar extent as without a HU delay (compare Figure 6G with Figure 1C). We also did not observe increased levels of pericentromeric cohesin in *iml3Δ* or *chl4Δ* mutants arrested in metaphase with sister kinetochores under tension after HU treatment (Figure 6H). These results rule out the possibility that a HU delay rescues cohesion at the pericentromeres of Ctf19 complex mutants by reversing the cohesin recruitment defect in these mutants. Rather, delaying S phase in Ctf19 complex mutants appears to overcome the failure of the late-arriving cohesin to become cohesive.

### Improved cohesion in *chl4Δ* and *iml3Δ* mutants in the *clb5Δ clb6Δ* background

**Figure 7:**
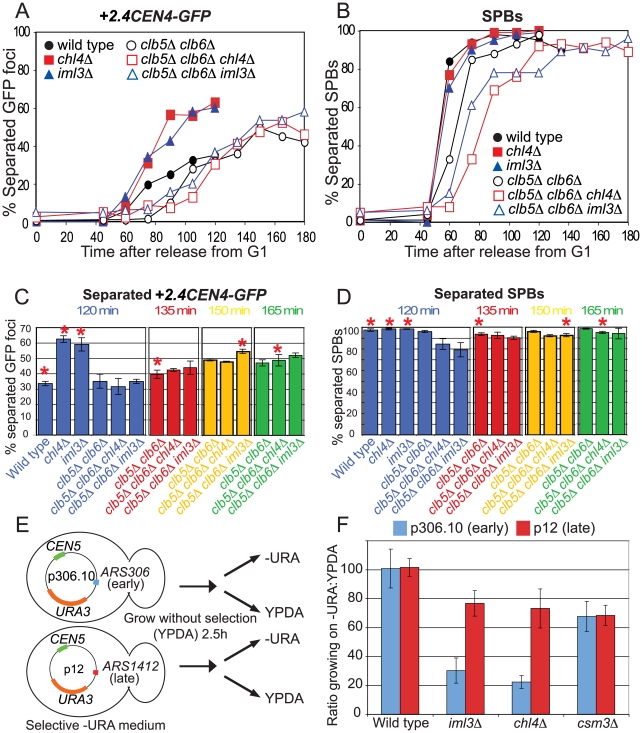
Replication timing affects cohesion establishment and plasmid maintenance in *chl4Δ* and *iml3Δ* mutants. (A–D) The frequency of *+2.4CEN4-GFP* dot separation in *clb5Δ clb6Δ* cells is not greatly increased by deletion of *IML3* or *CHL4*. Wild type (AM4643), *chl4Δ* (AM4644), *iml3Δ* (AM4647), *clb5Δ clb6Δ* (AM5351), *clb5Δ clb6Δ chl4Δ* (AM5426), and *clb5Δ clb6Δ iml3Δ* (AM5428) carrying *MET-CDC20*, *+2.4CEN4-GFP*, and *SPC42-tdTomato* were arrested in G1 with alpha factor and released into medium containing methionine. Percentages of separated GFP foci (A) and SPBs (B) are shown at the indicated time points for a representative experiment. After 120 min there were 5% (wild type), 3% (*chl4Δ*), and 1% (*iml3Δ*) anaphase cells, after which they escape from the metaphase arrest. Escape is delayed until 180 min in the *clb5Δ clb6Δ* cells, when anaphase cells account for 9% (*clb5Δ clb6Δ*), 3% (*clb5Δ clb6Δ chl4Δ*), and 9% (*clb5Δ clb6Δ iml3Δ*), respectively. Mean *+2.4CEN4-GFP* (C) and SPB (D) separation is shown from three (120, 135 min) or two (150, 165 min) experiments at the indicated timepoints with error bars representing standard error. (E,F) A plasmid with a late-replicating origin (p12) shows improved stability over a plasmid with an early-replicating origin (p306.10) in *chl4Δ* and *iml3Δ* cells. (E) Experimental outline is shown. (F). Ratio of colonies growing on selective medium that depends on the presence of the plasmid (*-URA*) compared to colonies growing on rich medium for wild type (AM1176), *iml3Δ* (AM3313), *chl4Δ* (AM3314), and *csm3Δ* (AM3194) strains transformed with the indicated plasmids.

As a further test of the ability of a replication delay to allow pericentromeric cohesion establishment in Ctf19 complex mutants, we examined the separation of *+2.4CEN4-GFP* dots in metaphase-arrested cells lacking the S phase cyclins, *CLB5* and *CLB6*, which are known to delay origin firing [49],[50] (Figure 7A–7D). FACS analysis revealed a 45 min delay in completion of DNA replication in cells lacking the S phase cyclins compared to wild type, *iml3Δ* or *chl4Δ* mutants (Figure S11). This delay in DNA replication was increased to 60 min and 75 min in *clb5Δ clb6Δ* mutants lacking *IML3* or *CHL4*, respectively (Figure S11), the reasons for which are unclear. We also observed a corresponding delay in spindle pole body separation in the *clb5Δ clb6Δ* background (Figure 7B). As before, *chl4Δ* and *iml3Δ* mutants separated *+2.4CEN4-GFP* foci rapidly and with a greater frequency than wild type (Figure 7A). As expected, due to the SPB separation delay, *+2.4CEN4-GFP* separation was delayed in cells lacking the S phase cyclins, however, deletion of *CHL4* or *IML3* did not increase the rate or frequency of separation (Figure 7A). This finding has to be interpreted with caution because *clb5Δ clb6Δ iml3Δ* and *clb5Δ clb6Δ chl4Δ* cells show an increased delay in spindle pole body separation (Figure 7B) as compared to *clb5Δ clb6Δ* cells, which could account for slower *+2.4CEN4-GFP* separation in these cells. However, taking the difference in the timing of SPB duplication into consideration, *+2.4CEN4-GFP* separation in *chl4Δ* or *iml3Δ* cells was reproducibly lower in the absence of the S phase cyclins, even though *clb5Δ clb6Δ* cells showed more separation than wild type, using these criteria (Figure 7C and 7D, compare time points with asterisks). Therefore, removal of the S phase cyclins appears to at least partially rescue the cohesion defect of *iml3Δ* and *chl4Δ* cells.

### A late firing origin rescues the stability of a centromeric plasmid in *iml3Δ* and *chl4Δ* cells

How does a replication delay allow the inefficiently loaded cohesin in Ctf19 complex mutants to become cohesive? As hypothesized above, it could restore the normal order of cohesin loading and replication fork passage, to allow cohesion generation through the usual replication-fork coupled mechanism in S phase. However, an alternative mechanism of global cohesion establishment has been described in G2 cells subjected to DNA damage [51]–[53]. Because HU is a DNA damaging agent and *clb5Δ clb6Δ* mutants have also been found to activate the DNA damage checkpoint [54], cohesion could be restored in *iml3Δ* and *chl4Δ* mutants through DNA damage-dependent cohesion establishment. To distinguish between the replication delay and DNA damage hypotheses we took advantage of the fact that some DNA replication origins are known to replicate early in S phase, whereas others replicate late. We used centromeric (*CEN5*) plasmids that carry a single origin that fires early (p306.10; *ARS306*) or late (p12; *ARS1412*) [55],[56]. Both plasmids also carry the *URA3* selectable marker, which allows growth on medium lacking uracil, enabling us to examine their ability to propagate in wild type, *chl4Δ*, *iml3Δ* and *csm3Δ* cells. Strains containing each of the plasmids were allowed to grow for 2.5 h in non-selective medium before plating onto selective (lacking uracil) and non-selective (rich) medium in parallel (Figure 7E). The ratio of colonies that are able to grow on medium lacking uracil compared to rich medium are given in Figure 7F. Although both plasmids were well maintained in wild type cells, they showed reduced stability in all three mutants. In the case of *csm3Δ* cells, we observed an equivalent decrease in stability of both the early and late-replicating plasmid, suggesting that replicating timing does not affect their ability to propagate in this mutant. Replication timing did however influence plasmid stability in *chl4Δ* and *iml3Δ* cells because the early-replicating plasmid displayed a greatly reduced stability compared to the late-replicating plasmid in these cells. Although we have not directly tested whether the decreased stability of the p306.10 plasmid in *iml3Δ* and *chl4Δ* mutants is the result of defective cohesion, these findings are consistent with the idea that pericentromeric cohesion establishment fails in cells lacking Ctf19 complex components because cohesin is not recruited prior to the passage of the replication fork.

## Discussion

### The Ctf19 complex and establishment of pericentromeric cohesion

In budding yeast, an approximately 50 kb region around the centromere, the pericentromere, is enriched for cohesin binding. Enhanced binding of cohesin in the pericentromere requires the ∼120 bp centromere sequence and is dependent on a functional kinetochore [13],[57]. Previous findings identified Ctf19 as being an important component of the kinetochore in this process [19]. We and others [58] have extended these findings and shown that the Ctf19 kinetochore subcomplex is an important mediator of cohesion establishment at the pericentromere. Since all single and double Ctf19 complex mutants show similar cohesion defects, the critical role of the Ctf19 and Mcm21 components in pericentromeric cohesion establishment may be to recruit the more peripheral components. However, our findings also indicate that Ctf19 complex components may have additional functions in chromosome segregation. In particular, Ctf19 and Mcm21 are unique in their requirement for chromosome segregation also during meiosis I. Whether this reflects a greater general requirement for these proteins in chromosome segregation (Table S1) or a specific role in, for example, generating linkages between homologs is unknown. However, Ctf19 components do not appear to play a major role in mediating kinetochore-microtubule attachments, since sister centromeres are pulled further apart in these cells. In addition, visualization of all kinetochores in Ctf19 complex mutants using Mtw1-GFP revealed no obvious attachment defect (JF and AM, unpublished observations). We note that human Mcm21 and Ctf19 proteins promote bipolar spindle assembly and chromosome congression, respectively [59] and that yeast Chl4 has been found to play a role in converting naked centromere DNA into an established centromere that is heritable [38]. Whether these functions of the Ctf19 complex are mediated solely through a role in cohesion establishment will be important to investigate in the future.

### Mechanism by which the Ctf19 complex promotes cohesin enrichment

How does the Ctf19 complex promote cohesin enrichment within the pericentromere? The simplest explanation is that the Ctf19 complex promotes the loading of cohesin at the centromere, which then spreads bidirectionally into the surrounding pericentromere. This could explain how the Ctf19 complex, which is localized within the ∼125 bp core centromere, is able to influence cohesin association over a much greater region. Support for this model comes from our finding that cohesin is detected at centromeric sites earlier in the cell cycle than at a pericentromeric site (Figure 5B–5D) and the observation that the Scc2 cohesin loader component is reduced at the centromere, but not other pericentromeric sites in the absence of *MCM21* [58]. However, Chl4, at least, may be able to influence cohesin association with the pericentromere independently of Scc2/4. We found that Chl4 is required for high levels of newly-synthesized cohesin to associate with the pericentromere during metaphase after the eradication of tension, a process that appears to be independent of Scc2 [23]. Although the functional relevance of metaphase-loaded cohesin remains unknown, these data provide evidence that the Ctf19 complex promotes cohesin association with the pericentromere throughout the cell cycle.

### Csm3 acts at a step after cohesin loading in pericentromeric cohesion establishment

We compared the contribution of Iml3-Chl4 to pericentromeric cohesion establishment with that of Csm3. The finding that cells lacking *IML3* and *CSM3* show synthetic cohesion defects suggests that these genes promote cohesion establishment through different pathways. Indeed, we observed no defect in cohesin association with the pericentromere in the absence of Csm3. This suggests that Csm3 facilitates cohesion establishment in a step after cohesin loading. Interestingly, Csm3 and its binding partner, Tof1, travel with the replication fork, and the Tof1-Csm3 complex is required for stable fork pausing at protein-DNA barriers including those at centromeres [5], [6], [45], [60]–[63]. Like Csm3, Tof1 has been implicated in cohesion [9],[11]. Tof1-Csm3 is conserved, being homologous to the Timeless-Tipin complex in humans and the Swi1-Swi3 complex in fission yeast [8],[9],[64]. Furthermore, the fission yeast Swi1-Swi3 complex is also required for the stabilization of stalled forks and efficient cohesion generation [8],[32]. Taken together, these observations indicate that the stalling and stabilization of replication forks may be the critical function of Csm3 in cohesion establishment. Indeed, we found that deletion of the helicase *RRM3*, which restores fork stalling to *csm3Δ* mutants [45], partially rescued the cohesion defect. Perhaps Tof1-Csm3 is required to maintain the association of essential cohesion establishment factors with the replisome upon encountering protein-DNA barriers. Such a hypothesis could explain why cells lacking *CSM3* show chromosome segregation defects in meiosis II, but not meiosis I. Perhaps there is a greater requirement for Csm3 in generating cohesion at protein-DNA barriers, such as centromeres, where kinetochore binding could impede replication fork progression. Alternatively, this could simply reflect the susceptibility of meiosis II to general cohesion defects due to the absence of arm cohesion [43].

### Cohesion establishment and DNA replication

Our analysis of cohesin association in a synchronized cell cycle showed that cohesin loads at centromeres early and in a Chl4-dependent manner. Given that centromeres replicate early in S phase [47], one important function of kinetochore-driven cohesin loading could be to ensure that cohesin is in place at centromeric regions prior to passage of the replication fork, thereby ensuring its incorporation into functional cohesion. In support of this model, allowing more time for cohesin loading by delaying DNA replication, either by HU treatment or deletion of the S phase cyclins, in *iml3Δ* and *chl4Δ* mutants improved pericentromeric cohesion. We cannot at present rule out that DNA damage-induced cohesion [51]–[53] is responsible for the restoration of pericentromeric cohesion in these cells. However, evidence that replication timing in the absence of DNA damage does play a role in centromere function in *iml3Δ* and *chl4Δ* cells, came from our observation that a *CEN5-*containing late-replicating plasmid is more stable than a similar early-replicating plasmid in *iml3Δ* and *chl4Δ* cells. Preliminary observations indicate that early and late replicating, but otherwise identical, *CEN4* plasmids behave in a similar manner (JF and AM, unpublished observations). It is interesting to note that while all centromeres replicate early in S phase, among centromeres, *CEN4* and *CEN6* replicate relatively late in their normal chromosomal context, whereas *CEN5* replicates early [47]. In wild type cells we saw the highest frequency of GFP dot separation with a probe close to *CEN5*. Whether this is due to the increased distance of the *CEN4* and *CEN6* probes from the centromere (2.4 kb and 4.5 kb, respectively, compared to 1.4 kb for *CEN5*), or an effect of their replication timing needs further investigation.

### A dedicated pathway for cohesion establishment at the pericentromere

A crucial role for pericentromeric cohesion has emerged during meiosis, where its protection during meiosis I ensures the fidelity of chromosome segregation during meiosis II [25]. However, it is becoming increasingly clear that pericentromeric cohesion plays specialized roles in mitosis too [19]. Being proximal to the site of microtubule attachment, it is required to resist spindle forces (Figure 2). Recent findings in fission and budding yeast have also demonstrated its importance in specifying the geometry of kinetochore-microtubule attachment [58],[65]. However, generation of cohesion at the centromere represents a considerable challenge, since kinetochore assembly generates a protein-DNA barrier to the replication fork whose passage is intimately linked to cohesion establishment. Furthermore, centromeres are known to replicate early during S phase [47], so that a mechanism must exist to ensure that cohesin is in place prior to passage of the replication fork. We propose that a two-step dedicated pathway of cohesion establishment involving the Ctf19 complex and Csm3 overcome these challenges to ensure the reinforcement of cohesion at the pericentromere. In the first step, the Ctf19 complex, through its Iml3 subunit, directs cohesin loading at the centromere, which subsequently spreads throughout the pericentromeric region. This dedicated pathway of cohesin loading is essential to ensure that cohesin is in place prior to passage of the replication fork early in S phase. Csm3 is crucial in this second step to ensure the integrity of the replisome and its association with cohesion establishment factors as it traverses the protein-DNA barrier that surrounds the centromere. The resultant enhanced cohesion in the vicinity of the centromere safeguards the fidelity of chromosome segregation. The Ctf19 complex and Csm3 are highly conserved. Whether such a mechanism of kinetochore-driven cohesion establishment operates in organisms where pericentromeric heterochromatin is known to attract cohesin [15]–[18],[66] is an important question for the future.

## Materials and Methods

### Yeast strains and plasmids

All strains used for meiotic experiments were derivatives of SK1 and all strains used for mitotic experiments were derivatives of W303. The *iml3Δ::KanMX6* and *chl4Δ::kanMX6* deletions were described in [27]. The *pCLB2-3HA-CDC20* fusion was described in [44]. The *REC8-3HA* and *SCC1-6HA* tags were described in [67] and [57], respectively. *pGAL-SCC1-3HA* was described in [4]. The *CHL4-6HA* and *IML3-6HA* tags were created using a PCR-directed method [68]. Other deletions were generated using genomic DNA from the yeast deletion collection [69] as template for PCR, followed by transformation. The *pMET3-CDC20* construct was constructed using a PCR based method as described by [70]. To create the *CEN6-GFP* strain, a 1 kb region to the right of *CEN6* was cloned into pRS306*tetO112* [42] and integrated into a strain carrying *tetR-GFP*, resulting in the generation of a GFP label 4.5 kb to the right of *CEN6*. The Spc42-tdTomato construct was described in [71]. Other GFP chromosome labels were described previously [21],[22],[42],[72]. Strains are listed in Table S2. Plasmids p12 and p306.10 were described in [56] and [55], respectively.

### Growth conditions

Growth conditions for individual experiments are given in the figure legends. All cultures were grown at room temperature unless otherwise stated. Meiosis was performed at 30°C as described in [73]. Drugs and alpha factor were removed by filtration, washing with at least 5 volumes of medium lacking sugar. Methionine was used at 8 mM and readded to 4 mM every hour. To depolymerise microtubules a mixture of benomyl (30 µg/ml) and nocodazole (15 µg/ml) were used and nocodazole (7.5 µg/ml) was readded every hour. Hydroxyurea was used at 10 mg/ml in liquid medium and 100 mM in plates.

### ChIP assay and quantification

ChIP was carried out as described by [14]. qPCR was performed in a 20 µl SYBR Green reaction using a BioRad iCycler machine. To calculate ChIP enrichment/input, ΔCT was calculated according to: ΔCT = (CT_(ChIP)_−(CT_(Input)_−logE (Input dilution factor))) where E represents the specific primer efficiency value. Enrichment/input value was obtained from the following formula: E^∧−ΔCT^. The average of 3 independent experiments for which qPCR was performed in triplicate are shown with error bars indicating standard deviation. We designed primers corresponding to cohesin-enriched (A1, A3) or cohesin-poor (A2) sites on chromosome arms as well as a centromeric site (C1) and several pericentromeric sites (P1–6), based on published ChIP data after microarray hybridization for the mitotic cohesin, Scc1 [23]. We were not able to analyze site C2 during meiosis since this primer set did not give a product in the SK1 strain background, presumably due to divergence from the published *S. cerevisiae* sequence. Sequences of primers are available on request. For the ChIP shown in Figure S1, primer sets were as described in [14] and Image J software was used to quantify ethidium bromide-stained gels.

### Chromosome loss assay and plasmid loss assay

To measure chromosome loss or plasmid loss, strains carrying the SUP11 artificial chromosome, or carrying the early or late replicating plasmids (p306.10 and p12), were grown overnight in minimal media lacking uracil (SD/-ura) before transferring to rich media (YPD) for 2.5–3 h. For the chromosome loss assay, approximately 2000–5000 cells were then plated out on YPD media. Loss of the artificial chromosome causes colonies to appear red. The percentage of sectored colonies that were at least half red, indicating loss in the first division after plating, were scored. To avoid amplification of earlier loss events, entirely red colonies were excluded from the analysis. For the plasmid loss assay, equal culture volumes totaling 1000–2000 cells were plated onto each of YPDA and SD/-URA and the percentage of colonies able to grow on SD/-URA, indicating retention of the plasmid, was calculated.

### Microscopy

Fixing cells for visualization of GFP-labeled chromosomes was performed as described by [67]. Indirect immunofluorescence methods were as previously described [74]. Microscopy was performed on a Zeiss Axioplan 2 microscope and for measurements of inter-*CEN* and –SPB distance, images were grabbed using a Hamamatsu ORCA-ER camera and analyzed using Zeiss Axiovision software. In the cohesion assay for mitosis, GFP dots were scored as separated if two dots were clearly visible in the same cell. A total of 200 cells were scored and all cells in the field were scored. To confirm metaphase arrest in each experiment, spindle pole body separation was analyzed either using Spc42-dtTomato or after anti-tubulin immunofluorescence or parallel samples. The leakiness of the *MET-CDC20* construct allowed spindle elongation in a few cells (usually<5% and never more than 10%) only at later timepoints (120 min) and was comparable in all strains in a given experiment. To score GFP dots in meiosis, cells were co-stained with DAPI to visualize nuclear morphology and 200 binucleate or tetranucleate cells were counted from the 5 and 8 h timepoints, respectively.

### FACS analysis

For flow cytometry, cells were fixed in 70% ethanol at 4°C over night. The cells were then treated with RNase over night, then digested with pepsin (Sigma). The cells were finally treated with propidium iodide (Sigma) to stain the DNA. Samples were briefly sonicated before analysis. FACS analysis was performed according to the manufacturer's instructions (BD FACS Calibur). FACS data were analyzed using CellQuest software.

### Western blotting

Samples for immunoblot analysis were prepared after TCA fixation as described by [75]. A goat Rad53 antibody (yC-19; Santa Cruz Biotechnology, inc.) was used at a dilution of 1∶1000.

## Supporting Information

Figure S1 Iml3 and Chl4 are required for cohesin enrichment at the pericentromere of chromosome V. (A) Locations of primers used for qPCR analysis on chromosome V. (B) qPCR analysis of ChIP samples used in Figure 1C. Mean values from two experiments are shown with error bars indicating standard deviation.
(0.57 MB EPS)

Figure S2 Iml3, Chl4, and Ctf3 are required for proper cohesion in the centromere. (A) Percentages of metaphase (open symbols) and anaphase (closed symbols) spindles are shown for wild type (black squares), *chl4Δ* (blue circles), and *iml3Δ* (red triangles) strains treated with DMSO for the experiment in Figure 2A after anti-tubulin immunofluorescence. (B,C) The percentages of separated spindle pole bodies (B) or *+2.4CEN4-GFP* foci (C) are shown at the indicated times after release from G1 for wild type (black squares), *chl4Δ* (blue circles), *iml3Δ* (red triangles), and *ctf3Δ* (grey diamonds) for 100 cells at each time point for the experiment shown in Figure 2D and 2E.
(0.95 MB EPS)

Figure S3 Similar cohesion defects during mitosis in Ctf19 complex mutants. (A–D) Similar frequency of *+2.4CEN4-GFP* separation in Ctf19 complex single and double mutants. Strains of wild type (AM4643), *chl4Δ* (AM4644), *mcm16Δ* (AM6148), *mcm22Δ* (AM6160), *chl4Δ* *mcm16Δ* (AM6195), *chl4Δ* *mcm22Δ* (AM6193), *ctf19Δ* (AM5786), and *mcm21Δ* (AM5788) mutants carrying *MET-CDC20*, *SPC42-dtTomato*, and *+2.4CEN4-GFP* were released from a G1 arrest into medium containing methionine to induce a metaphase arrest. The percentages of cells with separated spindle pole bodies (A,C) or *+2.4CEN4-GFP* foci (B,D) were scored at the indicated time points. (E,F) Ctf19 complex mutants exhibit a negligible cohesion defect at the *URA3-GFP* locus. Strains of wild type (AM1081) *iml3Δ* (AM3541), *ctf19Δ* (AM5814), and *mcm21Δ* (AM5812) mutants carrying *MET-CDC20* and *URA3-GFP* were treated as in (A–D). Spindle morphology after tubulin immunofluorescence (E) and separation of *URA3-GFP* foci (F) was scored at the indicated time points.
(1.30 MB EPS)

Figure S4 Reduced levels of Chl4 at the centromere in Ctf19 complex mutants during mitosis. Cycling cells of strains AM1176 (no tag), AM3276 (*CHL4-6HA*), AM4251 (*ctf19Δ* *CHL4-6HA*), AM4360 (*iml3Δ* *CHL4-6HA*), AM4544 (*mcm16Δ* *CHL4-6HA*), and AM5447 (*mcm21Δ* *CHL4-6HA*) were harvested for anti-HA ChIP followed by qPCR at the A1 and C2 sites as shown in Figure 1B.
(0.48 MB EPS)

Figure S5 Ctf19 and Mcm21 are required for accurate meiosis II chromosome segregation. The percentage of binucleate cells with the patterns of GFP dot segregation shown was determined after scoring 200 cells in the experiment shown in Figure 3A–3D. (A) Heterozygous dots; (B) Homozygous dots.
(0.66 MB EPS)

Figure S6 Iml3 and Chl4 are localized at the centromere in a Ctf19-dependent manner during meiosis. (A) Locations of primer sets along chromosome III used for PCR analysis of ChIP samples. (B) Chl4 localizes specifically to the core centromere in meiosis. Ethidium bromide-stained gels after PCR analysis of ChIP samples from diploid wild type strains carrying *CHL4-6HA* and otherwise wild type (AM3446), *mcm16Δ* (AM3803), *mcm22Δ* (AM3801), *ctf19Δ* (AM3773), or lacking an HA tag (strain AM1835) harvested 6 h after transfer into sporulation medium. These samples contained cells representative of all meiotic stages. (C) Iml3 localizes specifically to the core centromere during meiosis. Ethidium bromide-stained gels after PCR analysis of ChIP samples from diploid wild type strains carrying *IML3-6HA* and otherwise wild type (AM3441), *mcm16Δ* (AM3802), *mcm22Δ* (AM3800), *ctf19Δ* (AM3760), or lacking an HA tag (strain AM1835) treated as described in (B). (D) Quantification of ChIP PCR in (B). In each case, the signals were quantified and the ratio of ChIP to input (1∶500) PCR was calculated and shown as binding ratio. (E) Quantification of ChIP PCR in (C) performed as described in (D).
(3.29 MB EPS)

Figure S7 Csm3 is required for accurate chromosome segregation during meiosis and plays a non-overlapping role with Iml3. Cells of the indicated genotypes and in which both copies [(A,B); homozygous] or one copy [(C,D); heterozygous] of chromosome V marked with *URA3-GFP* were analyzed as described in Figure 1. Strains used in (A) and (B) were AM1603 (wild type), AM1903 (*iml3Δ*), AM3252 (*csm3Δ*), and AM3774 (*iml3Δ* *csm3Δ*). Strains used in (C) and (D) were AM107 (wild type), AM1904 (*iml3Δ*), AM3379 (*csm3Δ*), and AM3788 (*iml3Δ* *csm3Δ*).
(0.76 MB EPS)

Figure S8 Relationship between cohesin loading and DNA replication. FACS analysis of the experiment shown in Figure 5A–5E. Cells were analysed by flow cytometry to determine when the cells had replicated their DNA. For each sample, 15,000 cells were analysed.
(0.67 MB EPS)

Figure S9 DNA replication delay induced by HU treatment. FACS analysis of the experiment shown in Figure 6 (B–D). Cells were analysed by flow cytometry to determine when the cells had replicated their DNA. For each sample, 15,000 cells were analysed. (A) The cells were released from G1 arrest directly into media containing methionine. The red stars indicate the time after G1 release at which most cells have fully replicated genomes (2n). The numbers indicate the time after G1 release. (B) The cells were delayed in S-phase for 90 min by HU treatment, then released into media containing methionine. The blue star indicates the timepoint at which most cells showed fully replicated (2N) genomes. The numbers indicate the time following the release from the 90 min treatment of 10 mg/ml HU.
(2.98 MB EPS)

Figure S10 DNA damage checkpoint arrest and recovery after HU treatment. (A) Western blot showing Rad53 hyperphosphorylation upon HU treatment and restoration of faster migrating forms after release. Experiment was performed and strains used were as described in Figure 6B–6E, except samples were taken for Western blotting at the indicated timepoints and probed with anti-Rad53 antibody. (B) Sensitivity of wild type (AM1176), *iml3Δ* (AM3313), *chl4Δ* (AM3314), *ctf3Δ* (AM3958), *csm3Δ* (AM3194), and *rad53Δ* *sml1Δ* (AM6154) to HU. Serial dilutions of the indicated strains were spotted onto YPDA and plates containing 100 mM HU in parallel and incubated for 2 days at 30°C.
(7.24 MB EPS)

Figure S11 Delay in DNA replication in *clb5Δ* *clb6Δ* cells. FACS analysis for the experiment shown in Figure 7A–7D was performed as described in Figure S9 and in Materials and Methods. The asterisks indicate the completion of DNA replication in each strain.
(2.31 MB EPS)

Table S1 Chromosome loss rates.
(0.06 MB DOC)

Table S2 Yeast strains.
(0.18 MB DOC)
