# Supplementary material for: Establishment of Cohesion at the Pericentromere by the Ctf19 Kinetochore Subcomplex and the Replication Fork-Associated Factor, Csm3
Source: PLoS Genet. 2009 Sep 4;5(9):e1000629. doi: 10.1371/journal.pgen.1000629 (PMC2727958; doi:10.1371/journal.pgen.1000629)
Supplement: Table S1 — Chromosome loss rates. (0.06 MB DOC) [file pgen.1000629.s012.doc]

**Table S1. Chromosome loss rates**

|  | Experiment 1 | | Experiment 2 | | Experiment 3 | | mean loss/ division x 1000 | Standard deviation |
| --- | --- | --- | --- | --- | --- | --- | --- | --- |
|  | loss/ division x 1000 | *n* | loss/ division x 1000 | *n* | loss/ division x 1000 | *n* |
| Wild type | 1.7 | *4643* | 1.1 | *3667* | 1.2 | *3299* | **1.3** | 0.3 |
| *iml3Δ* | 38.2 | *5547* | 43.0 | *2141* | 38.6 | *2773* | **39.9** | 2.6 |
| *chl4Δ* | 77.9 | *5121* | 65.8 | *2403* | 68.7 | *2401* | **70.8** | 6.3 |
| *ctf3Δ* | 44.3 | *4643* | 33.3 | *2614* | 36.5 | *2524* | **38.0** | 5.7 |
| *mcm22Δ* | 60.7 | *5529* | 53.2 | *2614* | 56.7 | *2733* | **56.9** | 3.8 |
| *mcm21Δ* | 100.6 | *4827* | 105.4 | *1860* | 105.0 | *2047* | **103.7** | 2.7 |
| *ctf19Δ* | 117.1 | *4619* | 126.0 | *1920* | 130.8 | *1506* | **124.7** | 7.0 |
| *nkp1Δ* | 3.6 | *4955* | 2.6 | *2091* | 4.2 | *2795* | **3.5** | 0.8 |
| *nkp2Δ* | 4.3 | *6900* | 3.0 | *3478* | 2.2 | *3314* | **3.2** | 1.1 |
| *csm3Δ* | 64.9 | *4850* | 59.8 | *2681* | 58.7 | *2746* | **61.1** | 3.3 |
