# Supplementary material for: Establishment of Cohesion at the Pericentromere by the Ctf19 Kinetochore Subcomplex and the Replication Fork-Associated Factor, Csm3
Source: PLoS Genet. 2009 Sep 4;5(9):e1000629. doi: 10.1371/journal.pgen.1000629 (PMC2727958; doi:10.1371/journal.pgen.1000629)
Supplement: Table S2 — Yeast strains. (0.18 MB DOC) [file pgen.1000629.s013.doc]

**Table S2. Yeast Strains**

| Strain number | Relevant genotype |
| --- | --- |
| AM107 | *MATa/MATα LEU2::tetR-GFP/+ tetO-URA3/+* |
| AM436 | *MATa/MATα mcm21Δ/mcm21Δ LEU2::tetR-GFP/LEU2::tetR-GFP tetO-URA3/tetO-URA3* |
| AM437 | *MATa/MATα mcm21Δ/mcm21Δ  LEU2::tetR-GFP/+ tetO-URA3/+* |
| AM914 | *MATa MET-CDC20 LEU2::tetR-GFP tetO-CEN4 (+2.4CEN4-GFP*) |
| AM1081 | *MATa MET-CDC20 LEU2::tetR-GFP tetO-URA3* (*URA3-GFP*) |
| AM1105 | *MATa MET-CDC20 SCC1-6HA* |
| AM1145 | *MATa SCC1-6HA* |
| AM1176 | *MATa* W303 wild type |
| AM1603 | *MATa/MATα LEU2::tetR-GFP/LEU2::tetR-GFP tetO-URA3/tetO-URA3* |
| AM1835 | *MATa/MATα* SK1 wild type |
| AM1902 | *MATa/MATα chl4Δ/chl4Δ LEU2::tetR-GFP/+ tetO-URA3/+* |
| AM1903 | *MATa/MATα iml3Δ/iml3Δ LEU2::tetR-GFP/LEU2::tetR-GFP tetO-URA3/tetO-URA3* |
| AM1904 | *MATa/MATα iml3Δ/iml3Δ LEU2::tetR-GFP/+ tetO-URA3/+* |
| AM1905 | *MATa/MATα chl4Δ/chl4Δ LEU2::tetR-GFP/LEU2::tetR-GFP tetO-URA3/tetO-URA3* |
| AM2508 | *MATa MET-CDC20* |
| AM2511 | *MATa MET-CDC20 -30RTEL5-GFP* |
| AM3194 | *MATa csm3Δ* |
| AM3313 | *MATa iml3Δ* |
| AM3314 | *MATa chl4Δ* |
| AM3252 | *MATa/MATα csm3Δ/csm3Δ LEU2::tetR-GFP/LEU2::tetR-GFP tetO-URA3/tetO-URA3* |
| AM3276 | *MATa CHL4-6HA* |
| AM3375 | *MATa/MATα  pCLB2-CDC20/pCLB2-CDC20 REC8-3HA/REC8-3HA* |
| AM3377 | *MATa/MATα  iml3Δ/iml3Δ pCLB2-CDC20/pCLB2-CDC20 REC8-3HA/REC8-3HA* |
| AM3379 | *MATa/MATα csm3Δ/csm3Δ LEU2::tetR-GFP/+ tetO-URA3/+* |
| AM3411 | *MATa/MATα IML3-6HA/IML3-6HA* |
| AM3422 | *MATa/MATα  chl4Δ/chl4Δ pCLB2-CDC20/pCLB2-CDC20 REC8-3HA/REC8-3HA* |
| AM3441 | *MATa iml3Δ SCC1-6HA* |
| AM3442 | *MATa chl4Δ SCC1-6HA* |
| AM3446 | *MATa/MATα CHL4-6HA/CHL4-6HA* |
| AM3462 | *MATa bar1 CFIII URA3 SUP11* |
| AM3501 | *MATa MET-CDC20 CEN4-GFP chl4Δ* |
| AM3519 | *MATa MET-CDC20 URA3-GFP chl4Δ* |
| AM3522 | *MATa MET-CDC20 CEN4-GFP iml3Δ* |
| AM3541 | *MATa MET-CDC20 URA3-GFP iml3Δ* |
| AM3560 | *MATa/MATα pCLB2-CDC20/pCLB2-CDC20* |
| AM3597 | *MATa bar1 CFIII URA3 SUP11 iml3Δ* |
| AM3599 | *MATa bar1 CFIII URA3 SUP11 chl4Δ* |
| AM3601 | *MATa bar1 CFIII URA3 SUP11 ctf19Δ* |
| AM3604 | *MATa bar1 CFIII URA3 SUP11 mcm22Δ* |
| AM3661 | *MATa/MATα mcm22Δ/mcm22Δ LEU2::tetR-GFP/LEU2::tetR-GFP tetO-URA3/tetO-URA3* |
| AM3682 | *MATa bar1 CFIII URA3 SUP11 csm3Δ* |
| AM3684 | *MATa/MATα mcm22/ mcm22Δ LEU2::tetR-GFP/+ tetO-URA3/+* |
| AM3757 | *MATa csm3Δ SCC1-6HA* |
| AM3760 | *MATa/MATα IML3-6HA/IML3-6HA ctf19Δ/ctf19Δ* |
| AM3773 | *MATa/MATα CHL4-6HA/CHL4-6HA ctf19Δ/ctf19Δ* |
| AM3774 | *MATa/MATα csm3Δ/csm3Δ iml3Δ/iml3Δ LEU2::tetR-GFP/LEU2::tetR-GFP tetO-URA3/tetO-URA3* |
| AM3788 | *MATa/MATα  csm3Δ/csm3Δ iml3Δ/iml3Δ LEU2::tetR-GFP/+ tetO-URA3/+* |
| AM3798 | *MATa/MATα ctf19Δ/ctf19Δ LEU2::tetR-GFP/LEU2::tetR-GFP tetO-URA3/tetO-URA3* |
| AM3799 | *MATa/MATα mcm16Δ/mcm16Δ LEU2::tetR-GFP/LEU2::tetR-GFP tetO-URA3/tetO-URA3* |
| AM3800 | *MATa/MATα IML3-6HA/IML3-6HA mcm22Δ/mcm22Δ* |
| AM3801 | *MATa/MATα CHL4-6HA/CHL4-6HA mcm22Δ/mcm22Δ* |
| AM3802 | *MATa/MATα IML3-6HA/IML3-6HA mcm16Δ/mcm16Δ* |
| AM3803 | *MATa/MATα CHL4-6HA/CHL4-6HA mcm16Δ/mcm16Δ* |
| AM3811 | *MATa/MATα iml3Δ/iml3Δ chl4Δ/chl4Δ LEU2::tetR-GFP/LEU2::tetR-GFP tetO-URA3/tetO-URA3* |
| AM3887 | *MATa MET-CDC20 tetO--30RTEL5 LEU2::tetR-GFP iml3* |
| AM3942 | *MATa MET-CDC20 -tetO--30RTEL5 LEU2::tetR-GFP chl4Δ* |
| AM3948 | *MATa MET-CDC20 SCC1-6HA iml3Δ* |
| AM3950 | *MATa MET-CDC20 SCC1-6HA chl4Δ* |
| AM3958 | *MATa ctf3Δ* |
| AM4059 | *MATa/MATα ctf3Δ/ctf3Δ LEU2::tetR-GFP/LEU2::tetR-GFP tetO-URA3/tetO-URA3* |
| AM4084 | *MATa MET-CDC20 pGAL-SCC1-3HA* |
| AM4226 | *MATa MET-CDC20 SCC1-6HA CDC45-FLAG* |
| AM4251 | *MATa ctf19Δ CHL4-6HA* |
| AM4291 | *MATa MET-CDC20 SCC1-6HA CDC45-FLAG chl4Δ* |
| AM4360 | *MATa iml3Δ CHL4-6HA* |
| AM4544 | *MATa mcm16Δ CHL4-6HA* |
| AM4636 | *MATa bar1 CFIII URA3 SUP11 ctf3Δ* |
| AM4637 | *MATa bar1 CFIII URA3 SUP11 nkp2Δ* |
| AM4638 | *MATa bar1 CFIII URA3 SUP11 mcm21Δ* |
| AM4643 | *MATa MET-CDC20 LEU2::tetR-GFP tetO-CEN4 (+2.4CEN4-GFP*) *SPB42-tdTomato* |
| AM4644 | *MATa MET-CDC20 LEU2::tetR-GFP tetO-CEN4 (+2.4CEN4-GFP) SPB42-tdTomato chl4Δ* |
| AM4647 | *MATa MET-CDC20 LEU2::tetR-GFP tetO-CEN4 (+2.4CEN4-GFP) SPB42-tdTomato iml3Δ* |
| AM4679 | *MATa bar1 CFIII URA3 SUP11 nkp1Δ* |
| AM4683 | *MATa MET-CDC20 LEU2::tetR-GFP tetO-CEN4 (+2.4CEN4-GFP) SPB42-tdTomato ctf3Δ* |
| AM4717 | *MATa MET-CDC20 LEU2::tetR-GFP tetO-CEN4 (+2.4CEN4-GFP) SPB42-tdTomato csm3Δ* |
| AM4781 | *MATa/MATα nkp1Δ/nkp1Δ LEU2::tetR-GFP/+ tetO-URA3/+* |
| AM4927 | *MATa ctf3Δ SCC1-6HA* |
| AM4988 | *MATa/MATα nkp2Δ/nkp2Δ LEU2::tetR-GFP/+ tetO-URA3/+* |
| AM5104 | *MATa/MATα ctf3Δ/ctf3Δ LEU2::tetR-GFP/+ tetO-URA3/+* |
| AM5105 | *MATa/MATα mcm16Δ/mcm16Δ LEU2::tetR-GFP/+ tetO-URA3/+* |
| AM5107 | *MATa/MATα ctf19Δ/ctf19Δ LEU2::tetR-GFP/+ tetO-URA3/+* |
| AM5188 | *MATa MET-CDC20 LEU2::tetR-GFP tetO-CEN5 (+1.4CEN5-GFP) SPB42-tdTomato ctf3Δ* |
| AM5189 | *MATa MET-CDC20 LEU2::tetR-GFP tetO-CEN5 (+1.4CEN5-GFP) SPB42-tdTomato* |
| AM5249 | *MATa MET-CDC20 LEU2::tetR-GFP tetO-CEN5 (+1.4CEN5-GFP) SPB42-tdTomato iml3Δ* |
| AM5251 | *MATa MET-CDC20 LEU2::tetR-GFP tetO-CEN5 (+1.4CEN5-GFP) SPB42-tdTomato chl4Δ* |
| AM5312 | *MATa MET-CDC20 URA3-GFP csm3Δ* |
| AM5329 | *MATa MET-CDC20 CEN6-GFP SPB42-tdTomato* |
| AM5330 | *MATa MET-CDC20 CEN6-GFP SPB42-tdTomato iml3Δ* |
| AM5351 | *MATa MET-CDC20 LEU2::tetR-GFP tetO-CEN4 (+2.4CEN4-GFP) SPB42-tdTomato clb5Δ  clb6Δ* |
| AM5426 | *MATa MET-CDC20 LEU2::tetR-GFP tetO-CEN4 (+2.4CEN4-GFP) SPB42-tdTomato clb5Δ  clb6Δ  chl4Δ* |
| AM5428 | *MATa MET-CDC20 LEU2::tetR-GFP tetO-CEN4 (+2.4CEN4-GFP) SPB42-tdTomato clb5Δ  clb6Δ  iml3Δ* |
| AM5447 | *MATa mcm21Δ CHL4-6HA* |
| AM5533 | *MATa MET-CDC20 LEU2::tetR-GFP tetO-17.8CEN5 (-17.8CEN5-GFP) SPB42-tdTomato* |
| AM5537 | *MATa MET-CDC20 LEU2::tetR-GFP tetO-17.8CEN5 (-17.8CEN5-GFP) SPB42-tdTomato iml3Δ* |
| AM5542 | *MATa MET-CDC20 LEU2::tetR-GFP tetO-12.6CEN5 (-12.6CEN5-GFP) SPB42-tdTomato iml3Δ* |
| AM5545 | *MATa MET-CDC20 LEU2::tetR-GFP tetO-12.6CEN5 (-12.6CEN5-GFP) SPB42-tdTomato* |
| AM5551 | *MATa MET-CDC20 LEU2::tetR-GFP tetO-17.8CEN5 (-17.8CEN5-GFP) SPB42-tdTomato chl4Δ* |
| AM5560 | *MATa MET-CDC20 LEU2::tetR-GFP tetO-12.6CEN5 (-12.6CEN5-GFP) SPB42-tdTomato chl4Δ* |
| AM5564 | *MATa MET-CDC20 LEU2::tetR-GFP tetO-17.8CEN5 (-17.8CEN5-GFP) SPB42-tdTomato csm3Δ* |
| AM5569 | *MATa MET-CDC20 LEU2::tetR-GFP tetO-12.6CEN5 (-12.6CEN5-GFP) SPB42-tdTomato csm3Δ* |
| AM5786 | *MATa MET-CDC20 LEU2::tetR-GFP tetO-CEN4 (+2.4CEN4-GFP) SPB42-tdTomato ctf19Δ* |
| AM5788 | *MATa MET-CDC20 LEU2::tetR-GFP tetO-CEN4 (+2.4CEN4-GFP) SPB42-tdTomato mcm21Δ* |
| AM5796 | *MATa MET-CDC20 URA3-GFP csm3Δ  iml3Δ* |
| AM5808 | *MATa/MATα ctf3Δ/ctf3Δ chl4Δ/chl4Δ LEU2::tetR-GFP/+ tetO-URA3/+* |
| AM5809 | *MATa/MATα ctf3Δ/ctf3Δ chl4Δ/chl4Δ LEU2::tetR-GFP/LEU2::tetR-GFP tetO-URA3/tetO-URA3* |
| AM5812 | *MATa MET-CDC20 URA3-GFP mcm21Δ * |
| AM5814 | *MATa MET-CDC20 URA3-GFP ctf19Δ * |
| AM5974 | *MATa MET-CDC20 pGAL-SCC1-3HA chl4Δ* |
| AM6066 | *MATa MET-CDC20 LEU2::tetR-GFP tetO-CEN4 (+2.4CEN4-GFP) SPB42-tdTomato rrm3Δ  csm3Δ* |
| AM6068 | *MATa MET-CDC20 LEU2::tetR-GFP tetO-CEN4 (+2.4CEN4-GFP) SPB42-tdTomato rrm3Δ * |
| AM6145 | *MATa rad53Δ sml1Δ* |
| AM6158 | *MATa MET-CDC20 LEU2::tetR-GFP tetO-CEN4 (+2.4CEN4-GFP) SPB42-tdTomato mcm16Δ* |
| AM6160 | *MATa MET-CDC20 LEU2::tetR-GFP tetO-CEN4 (+2.4CEN4-GFP) SPB42-tdTomato mcm22Δ* |
| AM6193 | *MATa MET-CDC20 LEU2::tetR-GFP tetO-CEN4 (+2.4CEN4-GFP) SPB42-tdTomato chl4Δ mcm22Δ* |
| AM6195 | *MATa MET-CDC20 LEU2::tetR-GFP tetO-CEN4 (+2.4CEN4-GFP) SPB42-tdTomato chl4Δ mcm16Δ* |
